# Dissociation of Calcium Transients and Force Development following a Change in Stimulation Frequency in Isolated Rabbit Myocardium

**DOI:** 10.1155/2015/468548

**Published:** 2015-04-15

**Authors:** Kaylan M. Haizlip, Nima Milani-Nejad, Lucia Brunello, Kenneth D. Varian, Jessica L. Slabaugh, Shane D. Walton, Sandor Gyorke, Jonathan P. Davis, Brandon J. Biesiadecki, Paul M. L. Janssen

**Affiliations:** Department of Physiology and Cell Biology and D. Davis Heart Lung Institute, College of Medicine, The Ohio State University, 304 Hamilton Hall, 1645 Neil Avenue, Columbus, OH 43210-1218, USA

## Abstract

As the heart transitions from one exercise intensity to another, changes in cardiac output occur, which are modulated by alterations in force development and calcium handling. Although the steady-state force-calcium relationship at various heart rates is well investigated, regulation of these processes during transitions in heart rate is poorly understood. In isolated right ventricular muscle preparations from the rabbit, we investigated the beat-to-beat alterations in force and calcium during the transition from one stimulation frequency to another, using contractile assessments and confocal microscopy. We show that a change in steady-state conditions occurs in multiple phases: a rapid phase, which is characterized by a fast change in force production mirrored by a change in calcium transient amplitude, and a slow phase, which follows the rapid phase and occurs as the muscle proceeds to stabilize at the new frequency. This second/late phase is characterized by a quantitative dissociation between the calcium transient amplitude and developed force. Twitch timing kinetics, such as time to peak tension and 50% relaxation rate, reached steady-state well before force development and calcium transient amplitude. The dynamic relationship between force and calcium upon a switch in stimulation frequency unveils the dynamic involvement of myofilament-based properties in frequency-dependent activation.

## 1. Introduction

The force frequency response (FFR), or Bowditch effect, is one of the major physiological regulators governing alterations in contractile function of the heart [1, 2]. The FFR can occur in the absence of neurohumoral stimulation and is characterized by an increase in heart rate leading to an increase in contractile function [3, 4] and an acceleration of kinetics. These changes in force and kinetics result from alterations in cytosolic calcium levels as well as in the sensitivity of the myofilament to calcium [3–5]. During the FFR, increases in the frequency of pacing lead to an increase in the entry of calcium via the L-type calcium channel, subsequently increasing sarcoplasmic reticulum (SR) calcium load and release [3, 6]. A key characteristic of heart failure (HF) is a negative FFR, due to a decrease in calcium pump function and/or a decline in myofilament sensitivity [7–9]. Thus, the response in contractile function to changes in frequency is a diagnostic marker of the failing myocardium.

Although steady-state behavior of FFR is well investigated, the underlying regulatory mechanisms and the dynamic nature of this response are not fully understood. In nonfailing myocardium, changes in calcium and contractile function are known to involve multiple physical and chemical processes [2, 8]; however, the beat-to-beat variations in force and calcium homeostasis that occur during normal function are not well understood. Specifically, during the FFR there are changes in contractile function and calcium handling that happen on a timescale that does not easily lend itself to investigation as tools to assess myofilament responsiveness require minutes and/or the presence of steady-state conditions [10]. However, by taking a closer look at the temporal processes from one point of contractile steady-state (such as during rest) to another (such as during endurance exercise), we can potentially unravel changes in the relationship between force and calcium. This will allow us to then better characterize physiological mechanisms underlying the regulation of contraction and relaxation.

It has been shown by our lab and others that an increase in the frequency of pacing induces an increase in the calcium transient amplitude and a decrease in calcium transient duration [4, 6, 7]. These alterations in calcium handling eventually lead to molecular modifications within the myocyte [6]. Increases in frequency have also been shown to lead to increases in the phosphorylation state of many calcium handling channels and proteins such as the L-type calcium channel, troponin I (TnI), myosin binding protein C (MyBP-C), and phospholamban [3, 4, 11–14]. These alterations, together or separate, have the potential to either directly or indirectly lead to a change in myofilament calcium sensitivity and thus contractile kinetics.

Although much is known about the alterations in calcium handling when the heart is paced at different steady-state frequencies, the need for further understanding of the transitions between states is growing. Typically the heart beats at least once every second, with small and large changes in interbeat duration resulting from breathing and the level of physical activity and is not functioning at steady-state. Yet, most experimental designs require contractions to occur at a set frequency [10, 13]. Here we attempt to elucidate the effects of immediate changes in frequency on contractility and calcium transients at every beat. By determining what occurs during the stabilization of contractile force in relation to calcium handling we are able to better determine the functional characteristics of the calcium and force relationship.

Our results indicate that force and calcium are not changing in parallel and neither are force amplitude and contractile kinetics. Dissociation between the developed force and calcium transient has been documented following changes in muscle length at steady-state that indicate a myofilament-based process [15]. Our study highlights a disconnect between the intracellular calcium transient and the developed force during a dynamic frequency change, indicating the involvement of a myofilament-based regulation also during a change in the cardiac contractile steady-state.

## 2. Materials and Methods

### 2.1. Muscle Excision and Preparation

Male New Zealand white rabbits (2 kg, approximately 3 months old) were anesthetized with 50 mg/kg pentobarbital sodium (IV) and injected with 5,000 units/kg heparin. The investigation conforms to the* Guide for the Care and Use of Laboratory Animals* published by the US National Institutes of Health (NIH Publication number 85-23, revised 1996) and was approved by the local animal care and use committee.

The heart was rapidly excised and perfused retrogradely through the aorta with Krebs-Henseleit (KH) solution containing the following (in mmol/L): 120 NaCl, 5 KCl, 2 MgSO_4_, 1.2 NaH_2_PO_4_, 20 NaHCO_3_, 0.25 Ca^2+^, and 10 glucose (pH 7.4) in equilibrium with 95%O_2_/5%CO_2_ at 37°C. Additionally, 20 mmol/L 2,3-butanedione monoxime (BDM) was added to the dissection solution to stop the heart from beating and thus prevent cutting damage [16]. The effects of BDM after brief exposure have been found to be reversible [17]. Thin (to prevent core-hypoxia [18]) linear trabeculae from the right ventricular free wall were carefully excised and mounted between a force transducer and a micromanipulator. The muscle was bathed in a continuous flow of oxygenated 95%O_2_/5%CO_2_ KH solution (without BDM) at 37°C. The muscle was stimulated at 1 Hz, while calcium concentration was raised to 1.5 mM, and the muscle was stretched until maximal active force was attained. This length is comparable to maximally attained length* in vivo* at the end of diastole (around 2.2 *μ*m sarcomere length) [19]. The following protocols were run under isometric conditions with muscle length maintained at optimal length for maximal force production.

### 2.2. Twitch Protocol

Muscles were stabilized at 1 Hz for at least 10 minutes then subjected a rapid FFR interval change via a custom designed LabVIEW program, as previously published [10]. Briefly, muscle contractions were recorded at 1 Hz for between 30 and 120 seconds and were subjected to changes in frequency of pacing from 1 to 4 Hz (or 4 to 1 Hz). Muscles were then allowed to restabilize at the second frequency for an additional 1–10 minutes. Long periods of stabilization were avoided to prevent muscle run-down of twitch force [20]. Twitches and alterations in contractile kinetics, including developed force (*F*_dev_), time to peak force production (TTP), and 50% relaxation time (RT_50_), were analyzed using custom LabVIEW analysis software.

### 2.3. Calcium Transient

Intracellular calcium transient imaging was performed using Rhod-2 acetoxymethyl (AM) dye along with Olympus Fluoview 1000 confocal microscope in line-scan or XY-mode. Cytosolic calcium measurement was performed using Rhod-2 specifically for its brightness, photostability, and clarity of resolution [21–23]. This dye allows us to determine beat-to-beat alterations that are not visible using ratiometric non-AM dyes. Rhod-2 was excited by 488 and 543 nm and fluorescence was acquired at 550–650 nm [22].

Prior to data acquistion, muscles were incubated for 40 minutes in the dark at room temperature with Rhod-2. Rhod-2 AM was prepared in 2% pluronic, 0.2% cremphor, 0.1% TPEN, and KH solution. After incubation muscles were stimulated to contract at 1 Hz for an additional 5 minutes in Rhod-2 solution to ensure dye uptake into the muscle. Rhod-2 was then washed from the bath using the KH solution at 1.5 mM [CaCl_2_]. Fresh KH was then added and contractile force was allowed to stabilize for up to 20 minutes. After stabilization and visualization of proper dye loading, muscles were subjected to aforementioned twitch protocol.

Calcium alterations were measured at 10x magnification to determine global calcium transient and analyzed. Temporal dynamics in fluorescence of Rhod-2 was expressed as Δ*F*/*F*_*o*_, where *F* represents the fluorescence of Rhod-2 and *F*_*o*_ represents background fluorescence [22].

### 2.4. Myofilament Protein Preparation

To determine alterations in myofilament proteins (troponin T, troponin I, tropomyosin, myosin light chain 2, myosin binding protein C, and actin) phosphorylation status, individual trabeculae were flash frozen (<1 second) as previously described [4, 24] at 1 Hz, 4 Hz prior to stabilization, 4 Hz after stabilization for 1 minute, and 4 Hz after stabilization for 5 minutes. Muscles were homogenized by glass mortar and pestle in a solution containing the following: 75 mM KCl, 10 mM imidazole (pH 7.2), 2 mM EGTA, 2 mM MgCl_2_, 1 mM NaN_3_, 4 mM phosphocreatine, 1 mM DTT, and 1 mM benzamidine-HCl [25]. The solution was modified with the addition of 1% Triton depending on homogenization step. After homogenization protein pellets were resuspended in sample buffer containing the following: 6% SDS, 0.3% bromophenol blue, 30% glycerol, 150 mM Tris HCl, and 15 mM BME. Prior to loading, samples were heated to 80°C, vortexed, and centrifuged.

### 2.5. ProQ Phosphoprotein Stain and SYPRO Total Protein Stain

To determine key myofilament protein phosphorylation levels ProQ Diamond phosphoprotein gel (Life Technologies) and SYPRO Ruby total protein gel (Life Technologies) stains were used according to the manufactures instructions and similar to that previously described [4, 25]. Briefly, muscle myofilament preparations were fractionated by electrophoresis on a 12% 200 : 1* bis*-acrylamide Laemmli gel with a 4% 29 : 1* bis*-acrylamide stacking gel. ProQ Diamond followed by SYPRO Ruby protein gel staining was performed in dark at room temperature. Fluorescent imaging was done using a Typhoon 9410 imager (GE Healthcare) (ProQ Diamond: 532-nm excitation/580-nm BP 40 emission; SYPRO Ruby: 457-nm excitation/610-nm BP 30 emission) at a pixel size of 50 microns [14]. Resultant gel images were quantified by ImageQuant TL (GE Healthcare). Troponin T, tropomyosin, and myosin light chain 2 phosphorylation levels were normalized to SYPRO Ruby total protein stain levels for the protein of interest in the individual muscle while troponin I and myosin binding protein C phosphorylation levels were normalized to actin.

### 2.6. Data Analysis and Statistics

Calcium transient analysis was preformed through the use of ImageJ and Origin 7 software. Contractile data were collected and analyzed on- and offline using custom-written software in LabVIEW (National Instruments). Data are expressed as mean ± SEM unless otherwise stated. Data were statistically analyzed using ANOVA or Student's* t*-test (paired or unpaired) where applicable. A two-tailed *P* < 0.05 was considered significant.

## 3. Results

**Figure 1:**
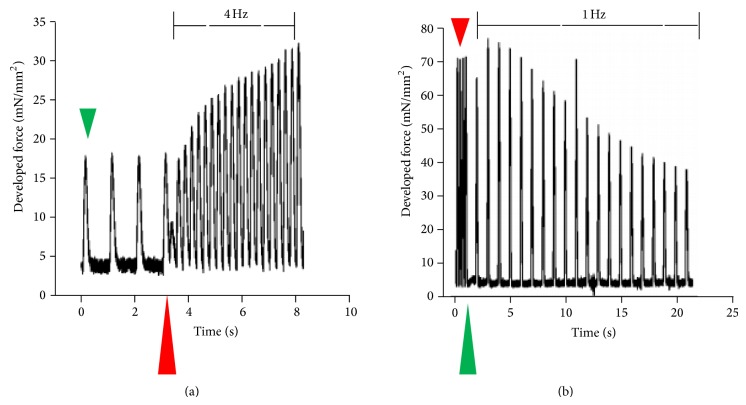
Beat-to-beat data output of twitches from an individual representative trabecula. Trabeculae from the New Zealand white male rabbit were excised from the right ventricular free wall and stabilized at either 1 Hz or 4 Hz. Muscles were perfused in KH with 1.5 mM CaCl_2_ at 37°C and were subjected to jumps in frequency from either (a) 1 Hz (green arrow) to 4 Hz (red arrow) with arrow marking moment of frequency change or (b) 4 Hz (red arrow) to 1 Hz (green arrow).

Frequency-induced changes in developed force and changes in contractile kinetics occur in several phases.  Figure 1 depicts a representative tracing of beat-to-beat alterations in developed force (*F*_dev_). The majority of changes in *F*_dev_ that occur on a beat-to-beat basis during a frequency jump from 1 to 4 Hz (Figure 1(a)) and 4 to 1 Hz (Figure 1(b)) occur within 20 beats during 1 or 4 Hz stabilization frequency. As shown, developed force of the first beat drops immediately following an increase in frequency. This drop is followed by a rapid increase in force production within 20 beats of the frequency change. Decreasing frequency from 4 to 1 Hz produces a transient increase in force production followed by a rapid decline. As can be seen in Figure 1, once the frequency is increased or decreased, there is an aberrant twitch immediately following the last twitch from the previous steady-state frequency.

**Figure 2:**
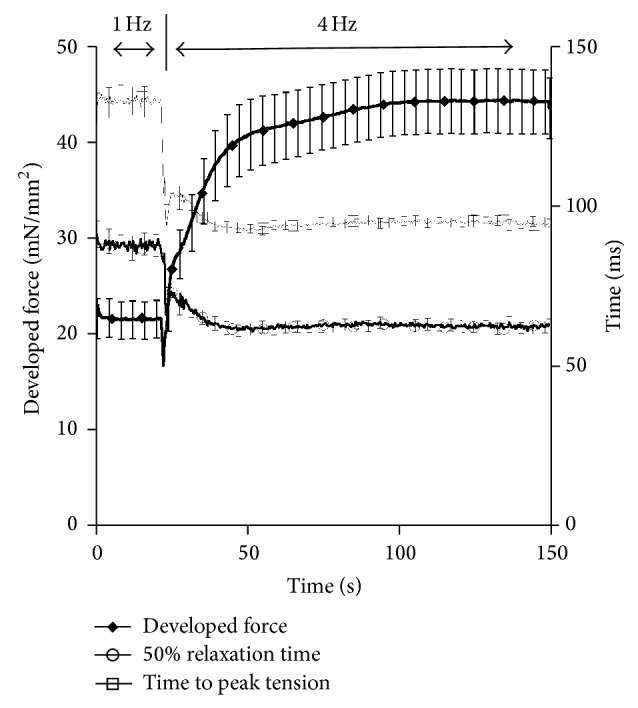
Kinetic tracings from muscles stimulated from 1 Hz to 4 Hz (*n* = 35). Developed force (*F*_dev_) increases rapidly and then gradually following an increase in pacing frequency. The switch in frequency is marked by a rapid decline in *F*_dev_ at approximately 60 seconds. Time to peak tension (TTP) and time to 50% relaxation (RT_50_) rapidly decline following an increase from 1 to 4 Hz pacing frequency and stabilize within 20 seconds of the frequency change.

Figure 2 depicts the alterations in contractile force and twitch kinetics that occur during the transition from 1 to 4 Hz. As the muscle continues at a higher pacing frequency the rapid increase in force production that occurs over the first 5–10 seconds (early phase) is followed by a slower phase (~1-2 minutes) in which the developed force gradually increases (late phase) and eventually stabilizes at the new steady-state. As more clearly shown in Figure 1, during the transition to 4 Hz, there is a rapid decline in force production of the 1st beat following a change in stimulation rate followed by a slower further increase until force stabilizes. Twitch kinetics, time to peak tension (TTP), and time from peak tension to 50% relaxation (RT_50_) measurements show a similar initial phase, but they reach the steady-state level prior to *F*_dev_ reaching the new steady-state.

**Figure 3:**
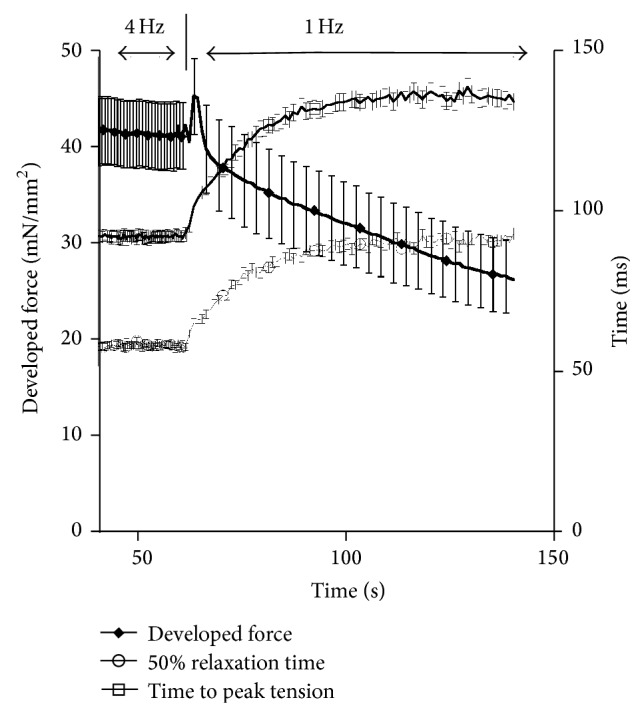
Kinetic response following a decrease in pacing frequency from 4 Hz to 1 Hz (*n* = 26). Developed force (*F*_dev_) decreases gradually following the initial change in pacing frequency. Prior to the decline in *F*_dev_ there is a rapid increase in force for approximately 5 seconds. Time to peak tension (TTP) and time to 50% relaxation (RT50) both increase following an increase in pacing frequency that stabilizes in approximately 30 seconds.

To determine the twitch dynamics that occurs during a decrease in pacing we reversed the previous protocol and changed the frequency from 4 Hz to 1 Hz. We then allowed the muscle to stabilize at 1 Hz. As depicted in Figure 3, as the pacing is immediately decreased from 4 to 1 Hz there is a rapid increase followed by a decrease in *F*_dev_. The stabilization at 1 Hz occurs much slower than the 4 Hz stabilization time previously presented. Additional studies on twitch kinetics during this drop in pacing frequency show a more gradual increase in TTP and RT_50_. As seen with increases in frequency, the twitch kinetics change rapidly with decreases in frequency and stabilize prior to the stabilization of force. These findings suggest additional alterations at either the calcium transient level or in myofilament calcium sensitivity. Thus, we set out to determine the effects of frequency on calcium transient amplitude.

**Figure 4:**
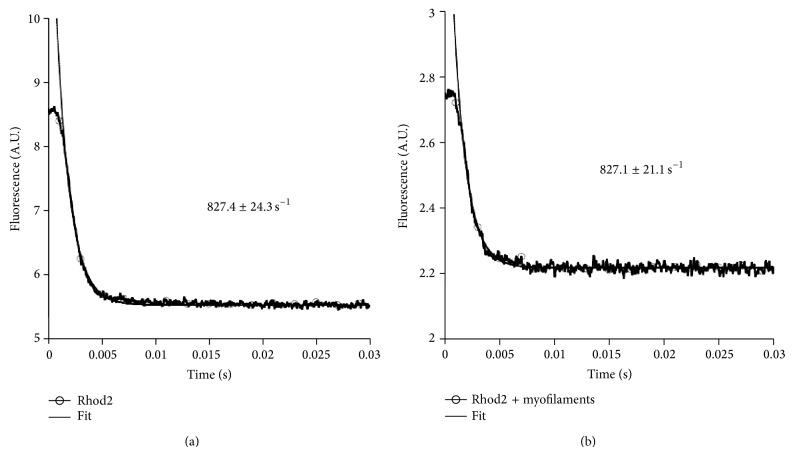
Stopped-flow analysis of the off-rate of Calcium from Rhod-2. Single experimental trace of determining the calcium off-rate, determined from a minimum of *n* = 10 runs in absence (a) or in presence (b) of myofilaments. Stopped-flow experiments were performed at 37°C.

In order to ensure that under our experimental conditions, most notably at 37°C, the dye can accurately track the calcium transient, the off-rate of the dye was investigated using a stopped-flow protocol as performed previously [15]. In Figure 4 the results of this verification are depicted in absence (a) and presence (b) of myofilaments.

**Figure 5:**
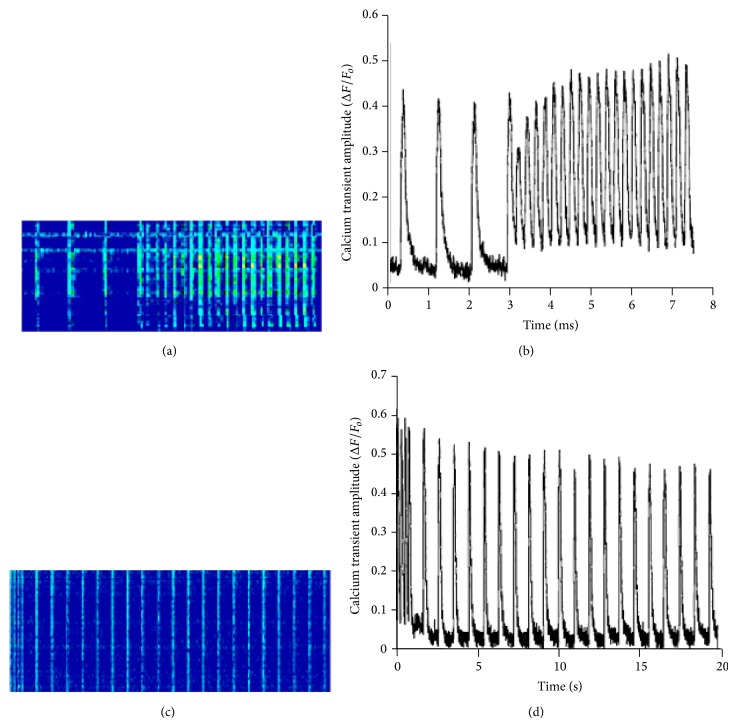
Line-scans of a Rhod-2 AM loaded isolated trabecula and changes in calcium amplitude during changes in pacing frequency from 1 Hz to 4 Hz and 4 Hz to 1 Hz. (a) Representative image of matrix line-scan of Rhod-2 AM loaded, isolated muscle, during frequency change from 1 Hz to 4 Hz and corresponding change (b) in calcium transient amplitude (Δ*F*/*F*_*o*_). Representative image of line-scan of Rhod-2 AM loaded isolated muscle (c) during frequency change from 4 Hz to 1 Hz and corresponding calcium transient amplitude trace (d) (Δ*F*/*F*_*o*_).

Confocal fluorescent microscopy line-scans of isolated trabeculae loaded with Rhod-2 AM (Figures 5(a) and 5(c)) show the changes in calcium transient in conjunction with changes in pacing frequency from 1 Hz to 4 Hz and 4 Hz to 1 Hz. Figures 5(b) and 5(d) depict these changes in a graphical representation of the microscopy line-scans. As the frequency is increased from 1 Hz to 4 Hz, the first beat is characterized by a depression of calcium transient amplitude due to incomplete SR reloading, followed by a gradual increase in the amplitude of the calcium transient along with an increase in peak twitch and end twitch calcium level. Analysis of Δ*F*/*F*_*o*_ show an immediate increase in diastolic calcium levels and a gradual increase in end twitch calcium levels (Figure 5(b)). Following a decrease in the frequency of pacing (from 4 Hz to 1 Hz), there is an immediate decline in the diastolic calcium level as well as in the end twitch calcium level (Figure 5(d)).

**Figure 6:**
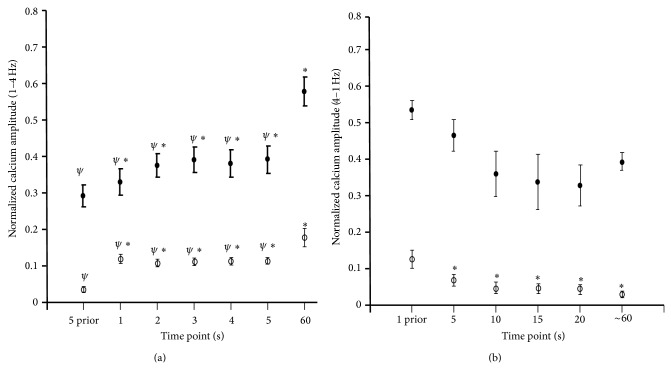
Normalized calcium transient amplitude during changes in pacing frequency. (a) Average relative calcium amplitude (*n* = 6) during the transition from 1 Hz to 4 Hz. Maximal and minimal calcium transient amplitudes were averaged for 5 seconds prior to the 4 Hz change in frequency, at the 1-, 2-, 3-, 4-, 5-, and 60-second time points following the frequency switch to 4 Hz. ∗ represents statistical significance from 5-second prior value and *ψ* represent statistical significance from 60-second value, *P* < 0.05. (b) Average relative maximal and minimal calcium transient amplitude recorded at 1 second prior to the 1 Hz frequency change and at 5, 10, 15, 20, and 60 seconds during the 1 Hz stabilization process (*n* = 4). ∗ represents a statistical significance from 1 second prior values. A *P* < 0.05 is considered significant.

Averages of the calcium transient alterations from 1 to 4 Hz show a drastic increase in the diastolic calcium level not necessarily due to changes in end twitch calcium levels (Figure 6(a)). The averages were taken at 5 seconds prior to the frequency change and at subsequent time points after the change in frequency. Additionally, once the muscle is stabilized (60-second time point), the end twitch and peak calcium levels are at their highest, as compared to all other earlier time points. To determine the effect of decreasing frequency on calcium transient, we examined the alterations in end twitch and peak calcium amplitude during the transition from 4 Hz to 1 Hz. In Figure 6(b), we looked at 4 beats (or 1 second) prior to the change in frequency, followed by 5, 10, 15, 20, and 60 seconds after the change to 1 Hz. During these times the functional changes during the frequency transition can be visualized and can thus be compared to the contractile function at steady-state. Once again we used the 60-second time point as a representative of the stabilized force and calcium transient amplitude. Here, we are able to show that with decreases in frequency there is a decline in end twitch and peak calcium amplitude maintained until the muscle has stabilized.

**Figure 7:**
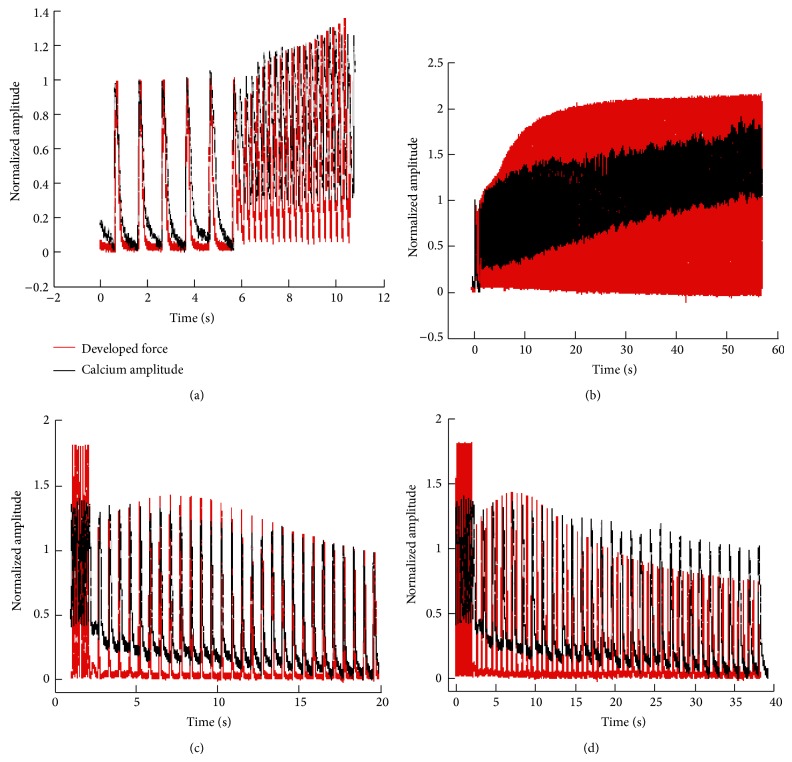
Force and calcium response during twitch after increases in pacing frequency. (a) Overlay of force and calcium tracings normalized to 1 Hz values during the transition from 1 Hz to 4 Hz (approximately 11 seconds). Twitch profiles and calcium transient changes are recorded for every contraction occurring in the trabecula during the brief transition from 1 Hz to 4 Hz. (b) Overlay of force and calcium tracings normalized to 1 Hz values. One twitch at 1 Hz is recorded followed by every twitch that occurs during the 4 Hz stabilization process (60 seconds total). (c) Normalized force tracings overlaid with normalized relative calcium amplitude transients of an individual isolated muscle during a frequency change from 4 Hz to 1 Hz (approximately 20 seconds). Tracings are normalized to 1 Hz stabilized values. (d) Overlay of force and calcium tracings normalized to 1 Hz values. Eight twitches at 4 Hz are recorded followed by every twitch that occurs during the 1 Hz stabilization process (40 seconds total).

In order to determine if calcium transient amplitude and *F*_dev_ would parallel each other through the early and late stabilization phases, we simultaneously measured *F*_dev_ and calcium transient amplitude during our frequency protocols. As shown in Figures 7(a) and 7(b), with increases in frequency there is no significant dissociation between end or peak calcium and *F*_dev_ during the early stabilization phase. Surprisingly, when taking an expanded look at the entire stabilization period, a rise in peak and end calcium occurs during the period at which the force has already reached a plateau. This phenomenon is not seen with a decreasing frequency of pacing (Figures 7(c) and 7(d)) suggesting a pertinent role for alterations in myofilament calcium sensitivity during stabilization.

**Figure 8:**
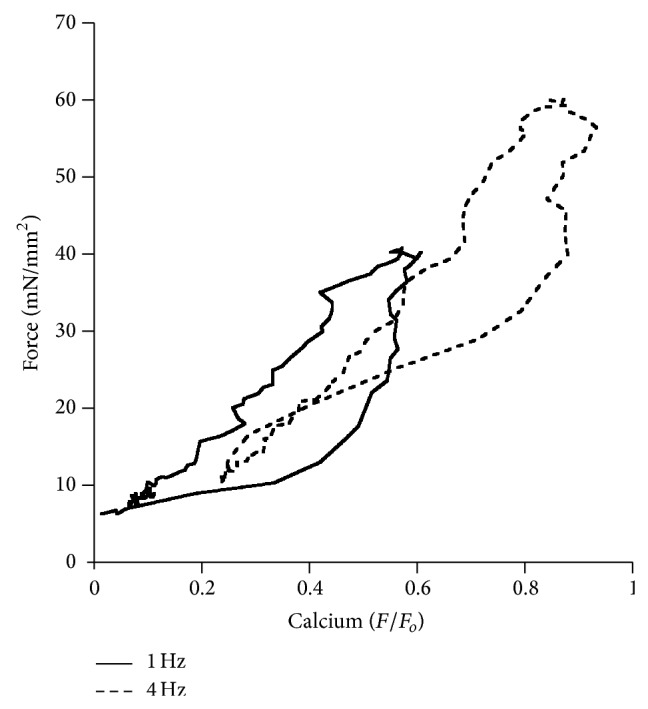
Phase-plane plot of force-calcium relationship. Force-calcium relationship at 1 Hz (solid line) and 4 Hz (dashed line) in a muscle paced stabilized at 1 Hz and thereafter paced and stabilized at 4 Hz.

Additionally, a phase-plane plot of the force-calcium relationship at 1 Hz and at 4 Hz (Figure 8) depicts an upward and leftward shift, indicative of a desensitization of the myofilaments at higher frequency.

**Figure 9:**
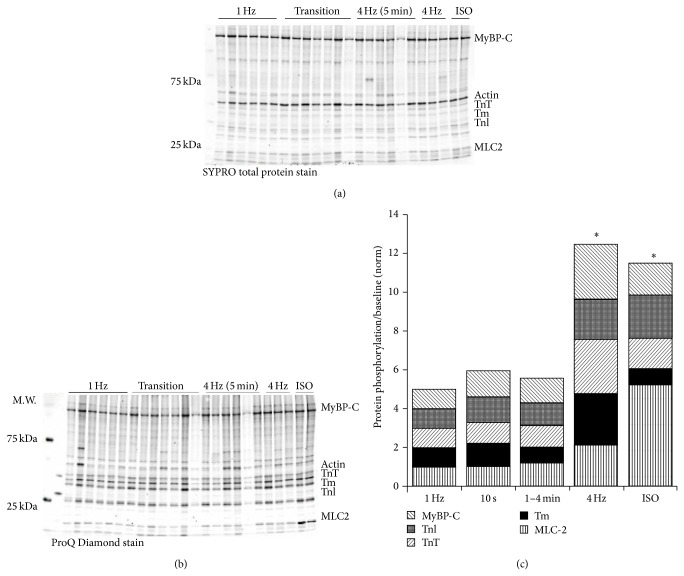
Alterations in phosphorylation during early and late frequency stabilization. Isolated trabeculae were frozen after stabilization at 1 Hz, up to 10 seconds following the switch from 1 Hz to 4 Hz, and 1 and 4 minutes after the switch from 1 to 4 Hz. Two trabeculae were also treated with ISO as a positive control for phosphorylation. (a) SYPRO Ruby total protein analysis of pertinent myofilament proteins. (b) ProQ Diamond analysis of key myofilament protein phosphorylation state. (c) Normalized (to 1 Hz) ratio of phosphorylated TnI and MyBP-C to total actin, and phosphorylated TnT, MLC-2, and Tm to total protein indicated a significant increase in cumulative phosphorylation at 4 Hz compared to 1 Hz but no significant changes during the early (10 seconds) and mid- (1–4 minutes) transition. ∗ represents a statistical significance from 1 Hz levels, *P* < 0.05.

In order to explain this dissociation between calcium amplitude and the force response, we attempted to identify posttranslational modifications associated with alterations in myofilament calcium sensitivity.  Figure 9 shows representative SYPRO Ruby total protein and ProQ Diamond phosphoprotein stained blots during 1 Hz stabilization (*n* = 6), 10 seconds after frequency-switch (*n* = 7), 1–4 minutes after frequency-switch (*n* = 6), 4 Hz steady-state (*n* = 3), and muscles stabilized at 1 Hz treated with 1 *μ*m isoproterenol (*n* = 2) to serve as a positive control. Key myofilament proteins (TnI, TnT, MyBP-C, Tm, and MLC2) were analyzed to determine alterations in phosphorylation status as compared to either SYPRO total protein for the protein of interest (TnT, Tm, MLC-2, TnI, and MyBP-C) or actin. Cumulative myofilament protein phosphorylation increased over time (*P* < 0.05, ANOVA), but the assay lacked the power to detect significance in individual protein phosphorylation levels (Figure 9(c)).

## 4. Discussion

The FFR has been utilized as a diagnostic tool to determine cardiovascular health. The FFR is characterized by a species-dependent increase in contractile force and acceleration of contractile kinetics following increases in the frequency of stimulation [5]. Calcium plays a critical role in the regulation of contractile function, and it is the calcium and force homeostatic relationship that is a critical regulator of the response to frequency on the heart [2]. In the past decade the focus was on understanding and utilizing the moderate variability in heart rate as a clinically diagnostic tool [26]. Heart rate variability (HRV) reflects the balance between the sympathetic and parasympathetic nervous systems [27]. Variability of the heart rate is intrinsic with increases in variability being considered as a positive marker of health [27, 28]. Our hope in this study is to utilize a simplified model of HRV to determine the mechanism regulating contractile stabilization from one steady-state frequency to another. Building a more in-depth understanding of the minute modifications that occur in a beat-to-beat regulated system is important to the development of potential HRV targeted therapeutic techniques.

Analyses of specific beat-to-beat force and calcium transient kinetics suggest a dissociation between the force production profile and contractile kinetics during the late phase of force stabilization. By determining the effectors that govern these alterations, we would eventually be able to stimulate or inhibit HRV in the failing myocardium. We here show that there is a disconnect between the changes in developed force and calcium amplitude in the late phase of force stabilization and that myofilament protein phosphorylation does not appear to play a significant role in the effects of early stabilization. This finding suggests a potential role for regulation at the level of cellular calcium entry and/or extrusion. In latter stages of stabilization, overall myofilament protein phosphorylation is increased, in accordance with earlier findings [4, 13, 14, 24]. The studies were performed at optimal length, where baseline phosphorylation of certain myofilament proteins may already be relatively high [29–31]. Also, note that overall phosphorylation of various sites may differentially impact function [32, 33], and further studies are needed to dissect these changes at the protein/specific site level.

Previous studies in our lab have shown that the interbeat duration can be a determinant of contractile function [24, 26]. Additionally, studies looking at the effects of previous beats have shown that interbeat duration impacts upon contractile function up to three beats prior to the beat of interest [24]. These effects have been studied in numerous species and point to a species-dependent interbeat duration relationship [34]. For this particular study we aimed to determine the difference between contractile kinetics and calcium transient on a beat-to-beat basis during alterations in frequency in the rabbit myocardium. Previously, studies have been unable to determine twitch and calcium transient characteristics following immediate changes in stimulated pacing frequency. Similar studies in isolated rat muscle have highlighted changes in peak active stress and correlated those changes to fluctuations in calcium levels [35]. However, here we are able to not only visualize the changes in maximal activation of force and calcium but also determine alterations that occur on twitch and calcium kinetics during every contraction. This technique enables the direct identification of how the characteristics of the calcium transient impact upon twitch kinetics. As shown above, when changing the frequency of pacing (from low to high), there is first an immediate and then gradual increase in force production. During decreased stimulation frequency, there is a characteristic increase in force production followed by a rapid force decline and then a more gradual decline as the muscle stabilizes at its new pacing frequency. It was our goal to determine the role of calcium transients and myofilament protein posttranslational modifications during the early and late phases of frequency stabilization. The kinetic profile of these muscles during the transition from low to high and vice versa parallels the changes in developed force.

Because most calcium handling studies are done in either mouse or rat individual myocytes [6] we aimed to gain insight into the global calcium transient in a multicellular preparation in a mammalian model that more closely resembles human calcium handling, which is different in many aspects from small mammals [36, 37]. Most calcium is circulated through the SR in rat and mouse (~90–98%) [6], thus concepts which focus mainly on SR function are valid. However, in humans the SR only accounts for ~70% of the calcium sequestering capabilities. Because of these intrinsic differences in calcium handling, mouse and rat models may progress faster through the early and latent phases during the frequency transition leading to faster stabilization. This suggests that HRV and frequency transition may not significantly affect the rodent physiology. For this study, we have utilized rabbit ventricular multicellular preparations at physiological temperature to aid in our understanding of human myocardial function and myofilament calcium sensitivity. Our study shows that during the early phase of the frequency stabilization the force response parallels the calcium response of both peak and end calcium levels. In the late phase (characterized by complete force stabilization) we observed a continual increase in end and peak calcium transient amplitude. This disconnect suggests an alteration in myofilament calcium sensitivity that is occurring late in the stabilization period. Upon a decrease in pacing frequency, there is a decrease in calcium transient amplitude followed by a more gradual decline as the muscle continues to stabilize. The peak and end calcium and force levels eventually decline in parallel as the muscle reaches steady-state. Together these findings suggest alterations in myofilament calcium sensitivity at higher frequencies that are later diminished as the muscle reaches steady-state at lower frequencies.

It was originally hypothesized that the latent stabilization phases were due to alterations in myofilament protein phosphorylation status which would account for changes in myofilament calcium sensitivity. However, proQ diamond phosphoprotein stain does not offer, with the experimental design of this study, to draw significance at least at the individual protein level, although overall the myofilaments are hyperphosphorylated at higher frequency. Our own as well as previous studies by others have highlighted the potential additive effect of myofilament protein phosphorylation [30, 33]. Additionally, changes in length have been associated with changes in myofilament protein phosphorylation [29]; thus utilizing optimal preload in the above experiments may induce phosphorylation to such a degree that differences induced by frequency are not significant. There is a clear role for the different calcium handling mechanism in the regulation of calcium transients during changing frequencies. The sodium-calcium exchanger (NCX) may be playing a role in the late phase, during which the muscle is stabilizing slowly and showing only gradual increases in contractile force [38]. NCX function has been shown to increase during the FFR [39, 40] and could account for the increases in peak and end calcium present during the late phase of frequency stabilization. Recent studies show S-glutathionylation of MyBP-C inducing a significant increase in myofilament calcium sensitivity [41]. Additionally, alterations through nitrosative and oxidative activity can also modify cytosolic calcium levels leading to changes in myofilament calcium sensitivity and force production [42]. For instance modification of the ryanodine receptor (RyR) by poly-S-nitrosylation increases calcium release potentially leading to a downstream effect of decreased calcium sensitivity at the myofilament level [43].

This study highlights a novel technique in which multicellular contractile kinetics and the corresponding calcium transient can be analyzed in the same muscle during a dynamic contractile protocol. Taken together, our results validate the importance of calcium transient amplitude when considering the rapid changes in pacing frequency, which occur endogenously. The disconnect between amplitude of the calcium and force transients further indicates a role of regulation at the myofilament level, other than through phosphorylation in the early stages of transition, in frequency-dependent activation and HRV.
